# Ultrasonography and magnetic resonance imaging changes in patients with polymyalgia rheumatica treated by tocilizumab

**DOI:** 10.1186/s13075-017-1499-2

**Published:** 2018-01-25

**Authors:** Anaïs Huwart, Florent Garrigues, Sandrine Jousse-Joulin, Thierry Marhadour, Dewi Guellec, Divi Cornec, Maelenn Gouillou, Alain Saraux, Valérie Devauchelle-Pensec

**Affiliations:** 1 Radiology Department, Cavale Blanche Hospital and Brest Occidentale University, Brest, France; 2 Rheumatology Department, Cavale Blanche Hospital and Brest Occidentale University, Brest, France; 3 INSERM UMR 1227, Laboratoire d’Immunothérapie et Pathologies lymphocytaires B, Labex “Immunotherapy, Graft, Oncology”, Brest Occidentale University, 29609 Brest Cedex, France; 4 Clinical Investigation Centre (CIC) 1412, Institut National de la Santé et de la Recherche Médicale (INSERM), Brest, France; 5 Radiology Unit, Hôpital de Cornouaille, 14 avenue Yves Thépot, F 29000 Quimper, France

**Keywords:** Polymyalgia rheumatica, Magnetic resonance imaging, Ultrasonography, Tocilizumab

## Abstract

**Background:**

This study assessed inflammatory changes using ultrasound (US) and magnetic resonance imaging (MRI) in patients taking tocilizumab for polymyalgia rheumatica (PMR).

**Methods:**

Eighteen patients were included in the prospective open-label TENOR study and received three tocilizumab infusions, without corticosteroids. B-mode and power Doppler US and MRI (T1 and T2-short time inversion recuperation weighted sequences) of the hips and shoulders were performed at weeks 0, 2, and 12. Subacromial, trochanteric, and iliopsoas bursitis and intraarticular glenohumeral and coxofemoral effusions/synovitis were scored from 0 to 3. Changes over time and US–MRI correlations were evaluated.

**Results:**

At baseline, the proportions of shoulders and hips with bursitis were 93 and 100% by MRI and 61 and 13% by US; and the corresponding proportions for intraarticular effusions/synovitis were 100 and 100% by MRI and 57 and 53% by US. Imaging findings did not improve during the first two treatment weeks. From baseline to week 12, bursitis improved significantly at all four joints by MRI (*P* = 0.005) and US (*P* = 0.029) and intraarticular effusions/synovitis by US only (*P* = 0.001). The proportion of abnormalities that improved by week 12 was 42% by MRI and 37% by US. MRI detected bursitis in a larger proportion of hips (73% versus 13%) and US in a larger proportion of shoulders (57% versus 28%), whereas no difference was found for intraarticular effusions/synovitis. At baseline, agreement between US and MRI findings was poor.

**Conclusions:**

US and MRI showed significant improvements in inflammatory lesions during tocilizumab treatment of PMR.

## Background

Polymyalgia rheumatica (PMR) is an inflammatory condition of unknown origin affecting people aged 50 years or older [1]. The main symptoms are inflammatory pain and prolonged morning stiffness of the shoulder and/or pelvic girdles [2]. Laboratory tests show severe systemic inflammation, and systemic corticosteroid therapy is rapidly effective [3–5].

Corticosteroids, however, induce numerous adverse events [6]. Tocilizumab (TCZ) is a humanized monoclonal antibody whose ability to block the IL-6 receptor inhibits the proinflammatory pathway. TCZ holds promise for the treatment of PMR [7–9].

Magnetic resonance imaging (MRI) and ultrasonography (US) are the main imaging techniques for detecting inflammation in and about the proximal joints [10–12] and, consequently, play a major role in evaluating PMR [13]. Subacromial-subdeltoid (SASD) bursitis, although not specific, is the imaging hallmark of PMR [14–16]. Hip joint bursitis and synovitis may explain the hip girdle symptoms experienced by patients with PMR [10, 17]. Another sign of proximal joint inflammation described in PMR is the presence of effusions in the glenohumeral and coxofemoral joints.

The follow-up of patients with PMR relies chiefly on serial determinations of the clinical PMR activity score (PMR-AS) and on laboratory markers for inflammation (C-reactive protein (CRP) and erythrocyte sedimentation rate) [18]. MRI and US are useful, however, to assess the treatment response. Both TCZ and corticosteroids effectively suppress the inflammatory process and would therefore be expected to normalize joint imaging findings. Studies using MRI [4, 16] and US [10, 19] found improvements during corticosteroid therapy. No similar studies are available with TCZ. Furthermore, the level of agreement between MRI and US findings is unknown.

We reported recently that TCZ monotherapy was effective in recent-onset PMR [20]. In 20 patients, the PMR-AS consistently fell below 10 by week 12, and no relapses occurred during follow-up (24 weeks). Similar results were obtained with a TCZ–corticosteroid combination [21]. The effect of treatment on MRI and US signs of inflammation was not assessed in these studies.

Here, our primary objective was to assess MRI and US changes during TCZ monotherapy for recent-onset PMR. Bursitis was assessed at the subacromial, trochanteric, and iliopsoas sites and intraarticular effusion/synovitis at the glenohumeral and coxofemoral joints. Our secondary objective was to assess agreement between MRI and US findings at baseline.

## Methods

### Patients

We conducted a prospective, cross-sectional, multicenter study in patients with PMR included in the TENOR study (Tocilizumab Effect iN pOlymyalgia Rheumatica) [20] (ClinicalTrials.gov NCT01713842) between September 2012 and May 2014. As described previously, TENOR was a double-blind study of TCZ, without corticosteroids, used to treat recent-onset active PMR. All patients met Chuang et al.’s criteria for PMR [22]. TCZ therapy was considered effective if 75% of patients had PMR-AS ≤ 10 by week 12. Three TCZ infusions were given, at weeks 0, 4, and 8. At one of the study centers (Brest), US and MRI of the shoulders and hips were performed at weeks 0, 2, and 12, using a standardized protocol. These imaging data form the basis for the study reported here.

### Imaging procedure

US was performed using an ESAOTE MyLab™ 60 machine (Esaote Medical, Saint-Germain-en-Laye, France) with an LA 523 4-13 MHz probe. B-mode and power Doppler were performed at each site. Bursitis was assessed at the SASD, trochanteric, and iliopsoas sites. The glenohumeral and coxofemoral joints were evaluated for synovitis and intraarticular effusion. Tenosynovitis of the long head of the biceps was sought. US abnormalities by B-mode or power Doppler imaging were graded using the OMERACT [23] semi-quantitative scale (0, no abnormalities; 1, mild; 2, moderate; and 3, marked) [24].

MRI was performed using a Philips Achieva 3 T machine (update dStream; Philips, Amsterdam, the Netherlands) with a 32-channel surface antenna. The following sequences were acquired at the shoulder and hip girdles: axial T1 turbo spin echo (T1 TSE), axial T2 short time inversion recuperation (T2 STIR), and coronal T2 STIR. For the T1 TSE sequence, the matrix was 828 × 420 and the field of view 53 × 32 cm at the shoulders; corresponding values were 720 × 404 and 46 × 31 cm, respectively, at the hips. Repetition time was 659 ms, echo time 20 ms, and slice thickness 4 mm. Fifty images were acquired in each plane. For the T2 STIR sequence at the shoulder, the matrix was 328 × 382 in the coronal plane and 524 × 210 in the axial plane; corresponding values at the hip were 356 × 420 and 468 × 264, respectively. Fields of view were 32 × 48 cm and 47 × 28 cm for coronal and axial imaging of the shoulder, respectively; corresponding values at the hip were 35 × 52 cm and 46 × 35 cm, respectively. Repetition and echo times at the shoulder were 4828 ms and 65 ms in the coronal plane and 2721 ms and 75 ms in the axial plane, respectively; corresponding values at the hip were 4979 ms and 60 ms in the coronal plane and 4677 ms and 65 ms in the axial plan. In the coronal plane, 22 slices were obtained with a thickness of 5 mm at the shoulders and 24 slices with a slice thickness of 4 mm at the hips. In the axial plane, 50 slices 4 mm in thickness were acquired at all sites.

**Fig. 1:**
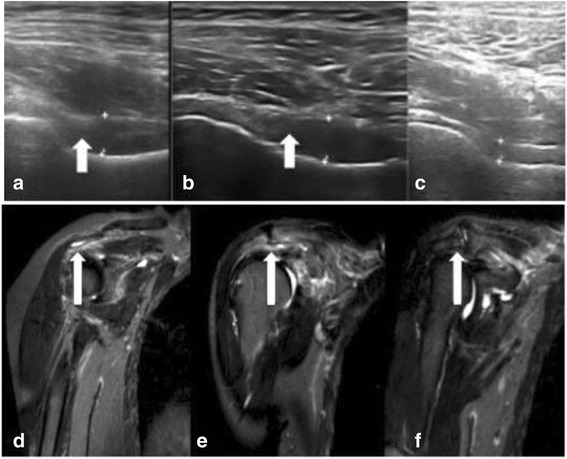
Improvements in ultrasound (US) and magnetic resonance imaging (MRI) abnormalities during tocilizumab therapy. (**1**) US: coxofemoral effusion at baseline (**a**), week 2 (**b**), and week 12 (**c**). Synovitis with synovial hypertrophy (arrows) scored 3 at baseline, 2 at week 2, and 0 at week 12. (**2**) MRI: subacromial bursitis at baseline (**d**, score = 3), week 2 (**e**, score = 2), and week 12 (**f**, score = 0)

Bursitis was defined as a high T2 signal and was assessed at the same three sites investigated by US. Effusion was also defined as a high T2 signal and was assessed at the glenohumeral joint, bicipital groove, and coxofemoral joint. For practical reasons, MRI was performed without contrast injection and a high T2 signal could therefore indicate synovitis and/or effusion. Abnormalities were scored using the already described OMERACT [23] semi-quantitative 0–3 scale (Fig. 1) [12, 16, 25, 26].

### Image analysis

US images were performed by two experienced observers (TM and SJ-J) specialized in osteoarticular US. MRIs were read by a senior radiologist (FG). All observers were blinded to patient data, and the MRI images for a given patient were read together but in random order. Before the study, intraobserver reliability was assessed by having the senior radiologist read 40 MRIs twice at an interval of 1 month. The kappa coefficient was 0.85 (0.72; 0.97).

The reference for imaging abnormalities was considered at joint level.

### Statistical analysis

Continuous variables were described as median and interquartile range (IQR) and categorical variables as *n* (%). Values at two time points were compared by applying the Wilcoxon signed-rank test for paired data. To evaluate sensitivity to change, we considered the sums of the two shoulder scores, of the two hip scores, and of the scores at both shoulders and hips.

Agreement between US and MRI findings at baseline was evaluated by computing Cohen’s kappa coefficient. Kappa values were interpreted as follows: < 0, no agreement; < 0.20, slight agreement; 0.21–0.40, fair agreement; 0.41–0.60, moderate agreement; 0.61–0.80, substantial agreement; and 0.81–1.00, almost perfect agreement.

## Results

### Setting and participants

Eighteen patients were included at the Brest center, seven women and 11 men, with a mean age of 71 years (range 58–83 years) and a mean body mass index of 28 ± 1 kg/m^2^. Mean serum CRP was 82 ± 16 mg/L (range 12–251) at baseline and 3 ± 2 mg/L (range 0.2–25.9) at week 12; corresponding values for the mean pain visual analog scale (VAS) score were 6.0 ± 0.5 (range 2.8–9.3) and 2.2 ± 0.4 (range 0.1–7.0). At baseline, 16 (89%) patients had shoulder-girdle pain and 16 (89%) had hip-girdle pain; corresponding numbers at week 12 were five (28%) and three (17%) patients, respectively. Mean PMR-AS was 38 ± 2 at baseline and 7 ± 1 at week 12. All patients improved clinically and achieved the TENOR end point of PMR-AS ≤ 10 by week 12.

### US and MRI evaluation

Technical problems precluded MRI in three patients. Another patient had MRI of the hips but not at the shoulders, due to severe pain. This left 28 shoulders and 30 hips with US and MRI data at baseline and at week 12 (Table 1). Bursitis defined as a score ≥ 1 was identified by MRI at the shoulders in 26 joints (93%) and at the hips in 30 joints (100%). Bursitis was less often detected by US, particularly at the hips (four joints, 13%). MRI was also more sensitive for detecting joint effusion/synovitis defined as a score ≥ 1 at both the shoulders and the hips. When bursitis or effusion/synovitis was defined as a score ≥ 2, the proportion of patients with the abnormality was smaller, but MRI remained more sensitive than US.

**Table 1:** Percentage of patients with abnormalities by MRI and US at baseline and at week 12

			Abnormality defined as a score^a^ ≥ 1		Abnormality defined as a score^a^ ≥ 2	
			Bursitis^b^	Effusion/synovitis^c^	Bursitis^b^	Effusion/synovitis^c^
Inclusion	MRI	Shoulders	93% (26/28)	100% (28/28)	64% (18/28)	71% (20/28)
		Hips	100% (30/30)	100% (30/30)	80% (24/30)	67% (20/30)
	US	Shoulders	61% (17/28)	57% (16/28)	21% (6/28)	21% (6/28)
		Hips	13% (4/30)	53% (16/30)	3% (1/30)	30% (9/30)
Week 12	MRI	Shoulders	93% (26/28)	100% (28/28)	46% (13/28)	64% (18/28)
		Hips	80% (24/30)	100% (30/30)	23% (7/30)	47% (14/30)
	US	Shoulders	36% (10/28)	50% (14/28)	0% (0/28)	18% (5/28)
		Hips	10% (3/30)	30% (9/30)	3% (1/30)	3% (1/30)

*MRI* magnetic resonance imaging *US* ultrasonography

^a^OMERACT Outcome Measures in Rheumatology score on a 0–3 scale, where 0 is normal

^b^Bursitis: subacromial bursitis at the shoulders and trochanteric and iliopsoas bursitis at the hips

^c^Shoulders: glenohumeral joint and long head of biceps; hips: coxofemoral joints

### Sensitivity to change

**Fig. 2:**
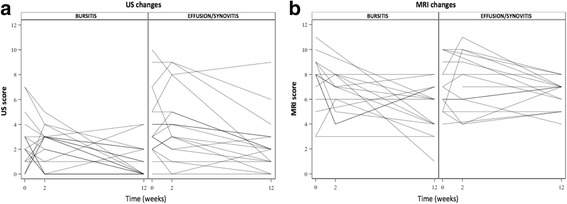
Changes in median ultrasound (US) and magnetic resonance imaging (MRI) scores during tocilizumab therapy. The bursitis and effusion/synovitis scores were computed by summing the scores for both shoulders and both hips. **a** MRI: bursitis (left) and effusion/synovitis (right). **b** US: bursitis (left) and effusion/synovitis (right). Individual patients are shown

Figure 2 and Table 2 report the changes in the median US and MRI scores with TCZ therapy. The only significant improvement from baseline to week 2 was for bursitis by MRI. In contrast, significant improvements occurred from baseline to week 12 for US bursitis (*P* = 0.029), US effusions (*P* = 0.001), and MRI bursitis (*P* = 0.005). Effusion/synovitis by MRI did not improve significantly from baseline to week 12 (*P* = 0.231). Improvements in SASD bursitis were better shown by US than by MRI.

**Table 2:** MRI and US findings at baseline and 2 and 12 weeks after starting tocilizumab therapy

				Wilcoxon test *P* value	
	Week 0	Week 2	Week 12	Week 0 vs week 2	Week 0 vs week 12
MRI (all four joints)					
Bursitis	8 (6–9)	7 (5–8)	6 (4–7)	0.109	0.005*
Effusion/synovitis	7 (5–9)	7 (6–9)	7 (5.5–7)	0.719	0.231
MRI (both shoulders)					
Bursitis	4 (2–4)	4 (3–4)	3.5 (2–4)	1.000	0.406
Effusion/synovitis	4 (3–5)	4 (3–5)	3.5 (3–4)	0.625	0.176
MRI (both hips)					
Bursitis	4 (3–6)	3 (2–4)	2 (1.5–3)	0.031*	< 0.001*
Effusion/synovitis	4 (2–4)	4 (3–4)	3 (2–4)	1.000	0.117
US (all four joints)					
Bursitis	2 (1–3)	3 (1–3)	0.5 (0–2)	0.575	0.029*
Effusion/synovitis	3 (2–5)	3.5 (2–5)	2 (1–3)	1.000	0.001*
US (both shoulders)					
Bursitis	2 (1–3)	2 (0–3)	0.5 (0–1)	0.542	0.018*
Effusion/synovitis	2 (1–3)	2.5 (1–4)	1 (1–2)	0.781	0.059
US (both hips)					
Bursitis	0 (0–1)	0 (0–0)	0 (0–0)	1.000	0.625
Effusion/synovitis	2 (0–3)	1 (0–2)	0 (0–2)	0.560	0.002*

Data are median (interquartile range)

*MRI* magnetic resonance imaging *US* ultrasonography

*Significant differences, Wilcoxon signed-rank test (*P* < 0.05)

### Changes in severity of imaging study abnormalities during TCZ therapy

Table 3 presents the proportions of US and MRI abnormalities whose score improved by at least 1 point from baseline to week 12. Overall, improvement ≥ 1 point occurred for 43/116 (37%) US abnormalities and 49/116 (42%) MRI abnormalities. The score remained unchanged for 59/116 (51%) US abnormalities and 58/116 (50%) MRI abnormalities. Worsening was uncommon, both for bursitis (5% by MRI and 17.5% by US) and for effusion/synovitis (10% by MRI and 7% by US). Improvements in hip bursitis were more often detected by MRI (22/30, 73%) than by US (4/30, 13%). At the shoulders, in contrast, improvements in bursitis were detected more often by US (16/28, 57%) than by MRI (8/28, 29%).

**Table 3:** Changes in severity of ultrasound (US) and magnetic resonance imaging (MRI) abnormalities from baseline to week 12

		Hips	Shoulders	Four joints
Bursitis^a^				
US	Improvement	13% (4/30)	57% (16/28)	34% (20/58)
	No change	77% (23/30)	18% (5/28)	48% (28/58)
	Worsening	10% (3/30)	25% (7/28)	17% (10/58)
MRI	Improvement	73% (22/30)	29% (8/28)	52% (30/58)
	No change	27% (8/30)	61% (17/28)	43% (25/58)
	Worsening	0% (0/30)	11% (3/28)	5% (3/58)
Effusion/synovitis^b^				
US	Improvement	47% (14/30)	32% (9/28)	40% (23/58)
	No change	50% (15/30)	57% (16/28)	53% (31/58)
	Worsening	3% (1/30)	11% (3/28)	7% (4/58)
MRI	Improvement	33% (10/30)	32% (9/28)	32% (19/58)
	No change	57% (17/30)	57% (16/28)	57% (33/58)
	Worsening	10% (3/30)	11% (3/28)	10% (6/58)

^a^Bursitis: subacromial bursitis at the shoulders and trochanteric and iliopsoas bursitis at the hips

^b^**S**houlders: glenohumeral joint and long head of biceps; hips: coxofemoral joints

Table 4 presents the proportions of US and MRI abnormalities that resolved fully; that is, whose score fell from ≥ 2 at baseline to ≤ 1 at week 12. The proportion of abnormalities that resolved was larger by US than by MRI.

**Table 4:** Number of findings of bursitis and effusion/synovitis, defined as a score^a^ ≥ 2, at baseline and at week 12, and percentages of abnormalities whose scores fell below 2 between baseline and week 12

		Baseline	Week 12	Grade < 2
MRI	Four joints	82/116	45/82	45% (37/82)
	Bursitis^b^	42/58	17/42	60% (25/42)
	Effusion/synovitis^c^	40/58	28/40	30% (12/40)
US	Four joints	24/116	5/24	71% (17/24)
	Bursitis^b^	9/58	0/9	100% (9/9)
	Effusion/synovitis^c^	15/58	5/15	67% (10/15)

*MRI* magnetic resonance imaging *US* ultrasonography

^a^OMERACT Outcome Measures in Rheumatology score on a 0–3 scale, where 0 is normal

^b^Bursitis: subacromial bursitis at the shoulders and trochanteric and iliopsoas bursitis at the hips

^c^Shoulders: glenohumeral joint and long head of biceps; hips: coxofemoral joints

### MRI–US agreement at baseline

Agreement was first studied with abnormalities defined as a score ≥ 1 by MRI, B-mode US, or power Doppler US (Table 5). Then, because MRI proved highly sensitive for detecting small abnormalities, we repeated the assessment using scores ≥ 2 by MRI and ≥ 1 by B-mode or power Doppler US. Agreement was slight at the hips but substantial at the shoulders.

**Table 5:** Agreement (kappa coefficient) between ultrasonography (US) and magnetic resonance imaging (MRI) at baseline

US/MRI	Bursitis^a^	Effusion/synovitis^b^	Bursitis^a^	Effusion/synovitis^b^
Right hip	0.03 (–0.04; 0.11)	0.31 (–0.002; 0.62)	0.12 (–0.05; 0.29)	0.29 (–0.21; 0.78)
Left hip	0	0.16 (–0.22; 0.54)	0.03 (–0.04; 0.11)	0.09 (–0.37; 0.55)
Right shoulder	0.28 (–0.04; 0.11)	0.20 (–0.05; 0.45)	0.19 (–0.23; 0.61)	0.51 (0.04; 0.99)
Left shoulder	–0.08 (–0.52; 0.37)	0.20 (–0.05; 0.45)	–0.40 (–0.74; –0.06)	**0.70** (0.32; 1)
Both hips	0.02 (–0.02; 0.05)	0.23 (–0.01; 0.42)	0.07 (–0.01; 0.16)	0.18 (–0.16; 0.52)
Both shoulders	0.11 (–0.16; 0.38)	0.20 (0.02; 0.37)	–0.14 (–0.50; 0.22)	**0.61** (0.30; 0.91)
Four joints	0.02 (–0.01; 0.15)	0.21 (0.06; 0.37)	–0.07 (–0.28; 0.13)	0.38 (0.15; 0.62)

Two left-hand columns: MRI and US abnormalities defined as a score ≥ 1. Two right-hand columns: MRI abnormalities defined as a score ≥ 2 and US abnormalities as a score ≥ 1

^a^Bursitis: subacromial bursitis at the shoulders and trochanteric and iliopsoas bursitis at the hips

^b^Shoulders: glenohumeral joint and long head of biceps; hips: coxofemoral joints

## Discussion

This is the first study of US and MRI changes in patients given TCZ without corticosteroids to treat recent-onset PMR. TCZ is being evaluated as a promising treatment for PMR. Using 18 F-FDG positron emission tomography–computed tomography, we demonstrated previously a significant but moderate decrease in the maximal standardized uptake value after 12 weeks of TCZ therapy [27].

In this study, patients underwent US and MRI at baseline and then 2 and 12 weeks into TCZ therapy. At baseline, MRI proved highly sensitive for detecting inflammatory lesions at the shoulders and hips. Thus, most patients had shoulder inflammation and all had hip inflammation. However, the clinical relevance of the minimal abnormalities that can be detected by MRI deserves discussion. High sensitivity may explain the persistence of effusions/synovitis after TCZ therapy by MRI but not by US. The US technique was less sensitive than MRI, showing SASD bursitis in 61% and hip bursitis in 13% of patients. Although SASD bursitis is recognized as a good diagnostic criterion for PMR [3], its frequency is controversial. Previous US studies found SASD in only18% [10] and 10% [28] of patients, in part because some patients were treated during the study and in part because US lacks sensitivity for detecting deep or thin abnormalities. In a previous study, of 57 untreated patients, 55 had abnormalities by US [12]. Finally, in a study of 50 patients with new-onset PMR, synovitis and/or effusion was consistently found in at least one joint [24].

Except for hip bursitis, the imaging study abnormalities failed to improve during the first 2 weeks of TCZ therapy. In rheumatoid arthritis, in contrast, early improvements have been reported with TCZ. However, in our study, TCZ was used alone, without corticosteroids, and at week 2 the patients had received a single infusion. A different treatment protocol might induce earlier improvements. Another possible explanation is that the inflammatory lesions in PMR may take time to respond to treatment. By week 12, the US and MRI abnormalities were significantly improved, in keeping with results in RA [29]. Thus, US and/or MRI may deserve consideration for objectively assessing the treatment response to 12 weeks of TCZ therapy. The serum CRP level is not a good monitoring tool, as it declines dramatically due to the mechanism of action of TCZ [30].

MRI and US proved sensitive to change during TCZ therapy for PMR. Nevertheless, the imaging study findings remained unchanged in a substantial proportion of patients, despite clinical improvements. Thus, the imaging study abnormalities may improve more slowly than the clinical manifestations. If imaging studies are used for follow-up, their timing should be given careful consideration. In a prospective 12-week study of 53 patients given corticosteroids to treat PMR, US was as sensitive, or more sensitive, to change compared to the clinical and laboratory markers of disease activity [19]. In a small minority of patients, the US and MRI findings worsened, despite clinical improvements. Worsening of imaging study findings may be related to intercurrent events (e.g., osteoarthritis flares) or may indicate limited reproducibility of image acquisition or interpretation.

At baseline, there was little agreement between US and MRI. Many patients had numerous inflammatory lesions by MRI but not by US. We therefore evaluated agreement between the two methods using a score ≥ 2 to define MRI abnormalities and a score ≥ 1 to define US abnormalities. In this analysis, agreement was substantial to nearly perfect (kappa = 0.61; 0.30–0.91) at the shoulders and poor at the hips. This difference is consistent with the larger number of US abnormalities detected at the shoulders than at the hips, which is probably due to the deeper position and, consequently, decreased accessibility of hip structures.

The main limitation of our study is its low statistical power due to the small sample size. We included patients enrolled at only one of the centers participating in a small, proof-of-concept study. Moreover, given the absence of a placebo arm, changes due to TCZ therapy cannot be differentiated from those due to the natural course of PMR. For practical reasons, the US studies were performed by two sonographers, but both were highly experienced and used to working together. TCZ is an emerging treatment for PMR, and our study provides the first data about its effects on imaging study abnormalities. Moreover, most of the earlier studies of imaging in PMR assessed MRI or US but not both [19, 31].

So, regarding our results, MRI could be useful in PMR for patients with atypical symptoms due to the high frequency of hip and shoulder abnormalities revealed by this procedure. Imaging can help to follow patients with residual pain after treatment.

## Conclusion

In patients given TCZ to treat recent-onset PMR, MRI and US showed improvements in bursitis and in effusions/synovitis in a substantial proportion of patients. These improvements were significant by week 12. In many patients, however, the imaging study abnormalities remained unchanged despite clinical improvements. US may be a good imaging technique for assessing the treatment response in PMR because it can show a complete regression of the lesions and also as it is noninvasive and widely available.

## Abbreviations

18 F-FDG: Fluorodeoxyglucose
3 T: 3 Tesla
CRP: C-reactive protein
IQR: Interquartile range
MRI: Magnetic resonance imaging
PMR: Polymyalgia rheumatica
PMR-AS: Polymyalgia rheumatica activity score
SASD: Subacromial-subdeltoid
STIR: Short time inversion recuperation
TCZ: Tocilizumab
TENOR: Tocilizumab Effect iN pOlymyalgia Rheumatica
TSE: Turbo spin echo
US: Ultrasonography
